# Cross-cultural adaptation of motivational interviewing for use in rural Nepal

**DOI:** 10.1186/s40359-021-00557-y

**Published:** 2021-04-01

**Authors:** Pragya Rimal, Sonu Khadka, Bhawana Bogati, Jamuna Chaudhury, Laxmi Kumari Rawat, Kumari Chhaya Bhat, Pramita Manandhar, David Citrin, Duncan Maru, Maria L. Ekstrand, Sikhar Bahadur Swar, Anu Aryal, Brandon Kohrt, Srijana Shrestha, Bibhav Acharya

**Affiliations:** 1 Nyaya Health Nepal, Kathmandu, Nepal; 2 Possible, New York, USA; 3 Department of Global Health, University of Washington, Seattle, WA USA; 4 Department of Anthropology, University of Washington, Seattle, WA USA; 5 Henry M. Jackson School of International Studies, University of Washington, Seattle, WA USA; 6 Icahn School of Medicine At Mount Sinai, Arnhold Institute for Global Health, New York, NY USA; 7 Department of Health Systems Design and Global Health, Icahn School of Medicine At Mount Sinai, New York, USA; 8 Department of Internal Medicine, Icahn School of Medicine At Mount Sinai, New York, NY USA; 9 Department of Pediatrics, Icahn School of Medicine at Mount Sinai, New York, NY USA; 10 School of Medicine, University of California San Francisco, San Francisco, USA; 11 Department of Psychiatry, Kathmandu Medical College, Kathmandu, Nepal; 12 Department of Psychiatry and Behavioral Sciences, George Washington University, Washington, DC USA; 13 Department of Psychology, Wheaton College, Norton, MA USA; 14 Department of Psychiatry, University of California San Francisco, San Francisco, USA

**Keywords:** LMICs, Motivational Interviewing, Nepal, Cultural adaptation, Global Mental Health

## Abstract

**Background:**

Motivational Interviewing (MI) has a robust evidence base in facilitating behavior change for several health conditions. MI focuses on the individual and assumes patient autonomy. Cross-cultural adaptation can face several challenges in settings where individualism and autonomy may not be as prominent. Sociocultural factors such as gender, class, caste hinder individual decision-making. Key informant perspectives are an essential aspect of cross-cultural adaptation of new interventions. Here, we share our experience of translating and adapting MI concepts to the local language and culture in rural Nepal, where families and communities play a central role in influencing a person’s behaviors.

**Methods:**

We developed, translated, field-tested, and adapted a Nepali MI training module with key informants to generate insights on adapting MI for the first time in this cultural setting. Key informants were five Nepali nurses who supervise community health workers. We used structured observation notes to describe challenges and experiences in cross-cultural adaptation. We conducted this study as part of a larger study on using MI to improve adherence to HIV treatment.

**Results:**

Participants viewed MI as an effective intervention with the potential to assist patients poorly engaged in care. Regarding patient autonomy, they initially shared examples of family members unsuccessfully dictating patient behavior change. These discussions led to consensus that every time the family members restrict patient's autonomy, the patient complies temporarily but then resumes their unhealthy behavior. In addition, participants highlighted that even when a patient is motivated to change (e.g., return for follow-up), their family members may not “allow” it. Discussion led to suggestions that health workers may need to conduct MI separately with patients and family members to understand everyone’s motivations and align those with the patient’s needs.

**Conclusions:**

MI carries several cultural assumptions, particularly around individual freedom and autonomy. MI adaptation thus faces challenges in cultures where such assumptions may not hold. However, cross-cultural adaptation with key informant perspectives can lead to creative strategies that recognize both the patient’s autonomy and their role as a member of a complex social fabric to facilitate behavior change.

## Background

Motivational Interviewing (MI) is a specific interaction strategy that enhances a person’s internal motivation to engage in healthy behaviors [1]. MI was developed in the United States in 1983 as a counseling technique to help patients with alcohol dependency [2, 3]. Over the years, it has been used for many medical and behavioral conditions [4].

MI emphasizes the partnership between the patient and the provider as a foundation of behavior change [5]. MI relies on cultural norms such as focusing on the patient as an individual and asserting the patients' autonomy over their behaviors. These assumptions may be more acceptable in western settings than in other cultural settings.

In social context where traditional gender roles are more prevalent, men are likely to voice their opinions and decide for themselves and their family members. Local religious and traditional practices can further reinforce these beliefs. To ensure the effectiveness of MI in such settings, it is important to adapt the concepts, protocols, and training materials [6]. Although MI manuals and guides have been translated to over 16 languages, much of the focus has been on immigrant populations within the US [7]. Research on cross-cultural adaptation of MI, especially in the Asian context, is limited [8]. Although numerous studies in cross-cultural settings report using MI, an in-depth discussion of the challenges and strategies in cross-cultural adaptation is rare in the scientific literature [9].

The Government of Nepal and the Ministry of Health and Population has integrated aspects of MI to assist people with alcohol and tobacco problems in its basic health package [10]. However, there are limited data in the literature describing the process of cultural adaptation of MI and using MI to aid behavioral change in non-western settings where the family has a more prominent role in making behavioral decisions for the patients [11, 12]. To the best of our knowledge, only one study has developed culturally tailored MI among South-Asian patients with hypertension living in Canada. The study provides culturally sensitive recommendations to improve medical adherence to blood pressure among patients with hypertension [9]. Here, we share our experience of translating and adapting MI concepts and skills to a local language and culture in rural Nepal. Cross-cultural adaptation in non-western settings includes several steps. One critical step is to incorporate the perspectives of key informants [7]. We report results from the adaptation experience, which is part of a larger study to train community health worker (CHW) in using MI to improve treatment adherence (e.g. taking medication and attending clinic appointments) for patients with chronic illnesses in rural Nepal [13, 14].

## Methods

### Setting

Our study site is Bayalpata hospital in Nepal’s far western district of Achham, one of the poorest regions of the country [15]. The hospital is managed by Nyaya Health Nepal, a nonprofit healthcare organization in close partnership with the government of Nepal [16] and Possible, a US-based nonprofit organization. The 50-bed general hospital employs over 150 staff and sees about 300 patients per day. The organization also mobilizes CHWs who provide care to patients in the nearby communities. We have described Nyaya Health Nepal’s CHW program in detail elsewhere [17]. Briefly, the CHW training is structured in three forms: [1] pre-service training, [2] program-based training, and [3] continuing education to conduct home visits to collect health related data (e.g., tracking new pregnancies) and assist patients and families engage in care (e.g., taking medications and returning to the healthcare facility for regular follow-up) by applying traditional methods of health education and advice giving. The CHWs use mHealth tools to facilitate home visits and to collect health-related data. The primary impetus for incorporating MI was driven by the gaps in interventions focused on advising and providing health education to patients, particularly those who were not interested in talking about behavior change or had initiated a healthy behavior (e.g., starting medications) but did not adhere to them.

### Intervention and data collection

Prior to this study, MI had not been formally tested in Nepal. We developed an MI training module in Nepali and field-tested it with five nurses who supervise CHWs. The nurses were good candidates as key informants for the field test because they operate at the interface of having received formal “western” medical training in nursing school while working exclusively with CHWs, who do not have formal healthcare training and are closer to the culture and context of the patients. The nurses had received at least Proficiency Certificate Level training, which includes three years of clinical education. All nurses available at the hospital were invited to the two-day field-test.

We used observation notes to describe challenges and experiences in cross-cultural adaptation. Observational structured note-taking is a well-established method to help generate and interpret data in health research [18, 19]. A bilingual psychiatrist, BA, was the dedicated note-taker who transcribed each reaction and question made by the participants. When a discussion ensued, the note-taker summarized the key points of the discussion. Key points of the discussion, including when consensus was achieved, were noted. The primary goal of structured note-taking was to catalog data on (a) conceptual items that were unclear to the participants; (b) concerns about lack of applicability in the local setting; (c) suggestions for adaptation for effective implementation and (d) case examples where MI would and would not work based on the participant's clinical experience with the local population. All participants were women from the local region, were under the age of 26, and had worked at Bayalpata Hospital for 5 to 46 months.

### Initial translation of MI concepts

The MI concepts used in the training module were initially translated from English to Nepali by BA, who is a bilingual psychiatrist with extensive experience in translating and cross-culturally adapting behavioral health and mental health concepts and interventions in Nepali [16, 20, 21]. The English source material was the most recent edition of Miller and Rollnick’s book on MI [22]. The first Nepali version developed by BA was discussed by BA and PR, who is a bilingual psychologist with experience in cross-cultural adaptation of mental health materials [16, 20, 21]. The two translators discussed all disagreements until arriving at a consensus, and the product of this process was used in the field test described in this study. The process of translation/back-translation and cross-cultural adaptation was based on the process described by Flaherty et al*.* [23], which we have used in prior studies [16, 20, 21]. The field-test training with the five nurses was delivered by PR during two six-hour sessions over two days. The training was disease-agnostic and used examples from various conditions. The training objective was to receive feedback on the content, which would be developed to train CHWs in the future. BA took structured observational notes based on participants’ responses in real-time by transcribing their comments.

### Data analysis

PR and BA reviewed and organized the notes into topics based on the primary competencies in MI [22]. This approach allowed the research team to adapt particular sections of the MI training and practice materials. A particular focus was on highlighting data that inform cross-cultural adaptation by situating MI’s basic assumptions within the implementation context of rural Nepal.

## Results

All participants (n = 5) attended the 12-h training. We present the data from the key informants based on an overview of MI followed by the core MI topics, as listed by Miller and Rollnick: 1) MI spirit (including partnership, autonomy, compassion, and evocation), 2) OARS (open-ended questions, affirmations, reflections, and summaries); and 3) change talk [22]. To accomplish effective juxtaposition, we have organized each sub-section by (1) describing each MI topic, (2) discussing our initial adaptation of the topics delivered to the participants, and (3) listing the participants’ responses.

### Translation and adaptation of key MI concepts

We translated the term “motivational interviewing” to “*ichhya badhaune paramarsha,*” that back-translates to “counseling to increase motivation.” Because the term “interviewing” can invoke a formal process of eliciting responses to structured questions, we avoided using that term. We also avoided the more commonly used Nepali term for motivation: “*prerena*” because it is closer to “inspiration.” As such, the back-translation would have been “counseling to increase inspiration,” which risks conveying that the counselor’s role is to make inspirational comments to the patient. In response to the overarching theme that the goal of MI is to enhance the patient’s motivation, participants agreed that it is preferable to increase the patient’s motivation rather than repeatedly telling the patient to change their behavior.

### MI spirit

“MI spirit” describes the philosophy behind MI in four overlapping domains: partnership, autonomy, compassion, and evocation. We translated “MI spirit” as “*biramilai herne dristikod*”, which back-translates to “the stance in viewing the patient.” Participants did not have any specific response to this term.

Next, we describe the results under the four sub-components of MI spirit.

### Partnership

MI emphasizes the partnership between the patient and the provider. Both have specific expertise, and for their relationship to be effective, the patient must be an active partner. While explaining the concept of partnership, we noted that both patients and providers have expertise. Participants’ initial response included, “but patients do not have expertise; the doctors and other providers are the experts.” As providers have gained formal knowledge about disease and medical treatments, they are regarded as experts and patients as learners. Because of this, participants initially suggested not using the word “expert” when describing the patient. This led to a discussion prompted by the question: ‘what kind of knowledge do patients have that the providers do not?’ Participants initially noted that some patients might know about their illness because they have read about it. One participant noted, “someone with diabetes may have read about it and thus may have expertise.” After continued prompting on other ways the patient could be an expert, participants noted, “the patient’s expertise is in knowing about their life and how the disease affects them,” and providers do not have that knowledge. Participants then noted that we are trying to challenge the traditional belief of what constitutes “expertise” and broaden the definition to include the patient’s knowledge about their illness and life experiences. After this consensus, participants agreed that this is an important reorientation and that the term *birami sanga pani bisesh gyan huncha* (“patients also have specific knowledge that providers lack”) should be retained.

### Autonomy

Autonomy is the principle that patients will and should make their own decisions. As part of the MI spirit, we used the Nepali term *swatantrata* (back-translates to “freedom”) to imply that patients are free to make their health decisions. Initial reaction from participants was that patients do not have full autonomy, and if they are not taking good care of their health, family members should use coercive measures. This led to a discussion of cases where coercion was used but wasn’t always successful.

One participant discussed a woman who smoked cigarettes: “her parents went through her belongings and threw away all the cigarettes, and kept doing it regularly until they could no longer find any cigarettes at home. They thought she had stopped smoking, but a few months later, they found out that she had been hiding cigarettes at her friend’s place and was smoking even more than she used to!” Participants shared similar stories about patients and family members where every time the patient’s freedom is restricted (e.g., hiding cigarettes), they comply temporarily and are more likely to go even further along the pathway of unhealthy behavior (e.g., finding yet another hiding spot and continuing smoking, likely even more than they used to). After this discussion, participants noted that patients, especially when addicted to substances, might do whatever they can to access the substances, and coercion often backfires. Participants indicated that due to the social context, some patients, particularly women, may not have the autonomy to make health decisions. A participant shared, “even if a woman wants to visit the hospital for her follow-up, her husband may not allow the visit if she has to take care of housework instead.” They discussed examples demonstrating that many patients have little autonomy. When asked what providers can do in such situations, participants wondered if they should use MI with the family member as well. Participants stated that using MI with the family members would help them understand what those family members care about. This process could help find alignment between what is best for the patient and what is important for her family members. For example, one participant suggested that having separate conversations given the differing motivations among the family members.

After discussion, participants noted that despite the cultural and contextual challenges, providers could still work with patients and their families to find common ground and use creative strategies to support the patient’s autonomy. A participant suggested that if the husband is concerned about the wife being by herself when traveling to the clinic for a follow-up appointment, then perhaps he would be more comfortable if she went with her friend/sister.

### Compassion

In MI, compassion goes beyond simply having a caring attitude and includes actively working in the patient’s best interest. We used the Nepali term “*karuna*” which means compassion to introduce the concept of compassionate care, and it resonated well with the participants. They relayed that some patients, particularly those not adherent to care, are often mistrustful of CHWs: “those patients accuse CHWs of trying to just get the patients to go to the hospital because they are employees of the hospital. They will say, “you always keep asking me to go to the hospital. That’s all you care about.” Those patients may not think CHWs are working in their interest. The participants welcomed the reorientation away from continuously reminding patients to accomplish a certain task and toward eliciting the patients’ view on describing what is best for them.

### Evocation

Evocation involves drawing ideas, experiences, information, and solutions from the patients. We used the Nepali terms “*birami bata sikne,*” which back-translates to “learn from the patients” to explain that our goal as providers is to have patients share ideas and solutions about their health. We further elaborated that evocation can be done by helping patients recognize what their obstacles are and what motivates them to overcome the obstacles.

Participants noted that they rarely use evocation. After learning about the other components of MI spirit, they said, “we usually train our CHWs to teach patients what to do. However, with everything we are learning about MI, evocation is important. If the patients say they want to do something, they will do it. We have been telling patients to do this, and that… and that approach hasn’t worked.”

### Core components of MI

The second section of the training included specific MI strategies to use while speaking with patients. We now describe the four components: Open-ended questions, Affirmation, Reflective listening, and Summaries. Often a fifth component, “information exchange,” is also included in this section, but we focused on the first four, as participants were previously not exposed to them.

### Open-ended questions

Open-ended questions set a non-judgmental tone and allow patients to provide explorative answers instead of answers such as “yes” or “no.” Some participants had heard about open-ended questions and knew these would help elicit more information from the patients. We used the Nepali term “*khula prashna sodhne*” which translates to “ask open questions.” Some participants were concerned that open-ended questions carry the risk of lengthening the visit. Others noted that this approach helped patients share their concerns and decreased the risk of patients thinking that CHWs are there with a specific agenda that does not accommodate patients' preferences.

### Affirmation

Affirmations in MI are statements of genuine appreciation highlighting the patients’ strengths, capabilities, and efforts rather than their problems and failures to change.

The concept of affirmations was thought to be particularly effective with patients who are the most resistant to change and often tell CHWs that they do not want to see them or even talk to them: “CHWs often ask us, ‘what should we do with this patient? He doesn’t want to talk to us about taking medications’, and now we know what they need to do.” The term “*samarthan garne*” (back-translates to “being supportive”) was used to describe that CHWs can affirm the patients’ strengths and efforts, rather than just telling them what they are doing wrong. Some participants noted that “supporting” could mean “agreeing with,” which could be problematic. If a patient says, “I do not want to speak with you,” the participants said that agreeing with that statement would make it impossible to get work done. However, after the trainer presented strategies to be supportive without agreeing (e.g., saying “you are someone who likes to speak their mind honestly”), the participants noted that affirming was different from agreeing and was a more effective strategy.

### Reflective listening

Simple reflection, such as repeating or restating patient responses, is a useful strategy in engaging with patients. Providers may use complex reflections by acknowledging patient ambivalence and ending the statement with target, healthy behavior, or by adding meaning and depth to patient statements. If a patient says they know they should go to the hospital but are afraid of people finding out about their disease condition, a complex reflection may be an effective response (e.g., “On the one hand, you are worried people may find out about your condition and you also know that you should visit the hospital”). We clarified how this reflection can steer the conversation towards engaging in healthy behavior while also helping the patient evoke their ideas and solutions.

We used the Nepali term “*shabdantaran garne wa doharaune*” (back-translates to “modifying some of the words or repeating them”). While discussing these techniques, participants agreed that acknowledging ambivalence and ending double-sided reflections with the target behavior are likely to be more effective. Participants responded, “it looks like MI was developed with a lot of research. We indeed tend to respond to the last thing that the other person says.”

To be effective, reflections are presented as statements (e.g., “I hear you saying that you have tried many times to quit smoking.”) and not as questions (e.g., “So are you saying that you have tried to quit smoking many times?”). The importance of reflections being statements rather than questions generated much discussion. During role-playing exercises to practice delivering reflections, when participants were supposed to respond with a simple reflective statement (e.g., “So you can’t imagine ever stopping smoking.”), they often ended the statements with “right?”, “ok?” or an upward inflection, turning the reflection from a statement to a question. We emphasized that reflections that are not statements will no longer be reflections but will become questions. Participants were concerned that such statements would seem impolite. They noted that tag questions are commonly used in polite conversation and sometimes to solicit agreement, there is a risk that avoiding tag questions can make the speaker sound presumptuous.

However, participants noted that asking a lot of questions can make the patient feel guarded and defensive, whereas using statements facilitates a more open and natural conversation. We then suggested that reflective statements can be thought of as a way to convey, ‘I understand and see what you are saying’ rather than an attempt to question what the patient is saying. Participants then practiced the same role-playing exercises, but when they made reflective statements, they were asked to mentally say “umm-hmm” while nodding their heads. Participants noted that this approach helped them maintain reflections as statements (i.e., not using question tags) while also conveying politeness and appropriateness because they felt that they were demonstrating understanding rather than presumption.

### Summaries

The strategy of summarizing patient statements can help providers retain and understand information while conveying that the patients are being heard and understood.

We used the term “*saransha sunaune”* which back translates to “convey the summary.” Participants were familiar with this strategy and agreed it was an important technique in confirming what patients said and would help in maintaining, and sometimes switching, the focus of the conversation.

### Change talk

Change talk includes patient statements favoring the direction of change towards healthy behavior (e.g., a patient saying, “despite all the reasons, I do think it is important to take medications.”). In MI, the goal is to approach the patient with the MI spirit and use OARS to elicit change talk from the patient because such statements have been shown to predict actual behavior change [22]. We used the Nepali words “*pariwartan ka shabdaharu*” (back translates to “words about change”).

Participants said the concept made intuitive sense and that the more time patients spend talking about change, the higher the likelihood that those patients will actually change. Participants noted that, in the past, CHWs would be frustrated when the patient didn’t make a clear commitment. They would often keep asking “you will take your meds, right?”. Upon further discussion, participants noted that this approach could help CHWs recognize the goal of their visit is to simply elicit change talk, even if it is part of an ambivalent response, rather than expecting the patient to make firm, unequivocal commitments to change their behavior.

### Stages of change

Traditionally, MI has been associated with the stages of change model where patients move through different stages in the direction of healthy behavior. The five stages of change are pre-contemplation (not interested in change), contemplation (considering change but still ambivalent), preparation (decided to change but haven’t acted on it), action (behavior change has taken place), and maintenance (behavior change is sustained) [24]. This may happen in a nonlinear manner, and patients often go through each stage multiple times in the change process [25].

We introduced the stages of change model as it helped providers recognize that the visit goals may need to be adjusted to the patient’s readiness for change. Participants shared that the staged approach to assessing patients was helpful. To describe the various stages of change, we used a fork metaphor in the road where one road is paved and another is unpaved while there is a thick forest between them. The metaphor was acceptable and easily accessible. If a person has traveled far along the dirt road (i.e., engaged in unhealthy behaviors, such as smoking, for a long time), trying to force them to go on the paved road (i.e., engaging in healthy behaviors, such as living a smoke-free life) would be met with resistance because the patient knows the dirt road best. It can be intimidating to cross over to the paved road because you need to travel through the thick forest. This stage, where the patient is happy being on the dirt road, despite the provider seeing that as a bad choice, was associated with the pre-contemplation stage, where the patient seems to have no interest in leaving the dirt road (and the unhealthy behaviors). Participants said that CHWs frequently tell them about patients at this stage. Such patients show no interest in change and often yell at CHWs who visit patients for follow-up and try to advise them on the importance of healthy behavior. After the analogy with the dirt road, participants noted that they could empathize with patients wanting to stay on the unhealthy path because it can be scary to move to the other road, which is unfamiliar.

The metaphor was further used to explain the patient’s ambivalence as they stand at the crossroads. Participants agreed that a more reasonable goal for someone who has traveled far along the dirt path (i.e., pre-contemplation) is to bring them to the crossroads first (contemplation). Only after getting to the crossroads could the patient reasonably prepare to take the paved road (preparation) and continue walking on it (action and maintenance).

Participants found this approach helpful and noted that they had been using the same techniques of educating and advice giving to all patients, regardless of where the patient was in the stages of change model. They liked the idea of using different strategies and setting different goals, depending on the patient’s readiness to change (Table 1).

**Table 1:** List of MI concepts and strategies, cross-cultural concerns addressed during field-testing, and the adaptation of the finalized MI concepts and strategies

MI concepts or strategy	Cross-cultural concern	Adaptation
Motivational Interviewing (MI)	A commonly used word for Motivational in Nepali, *prerena,* is closer to “inspiration.” Similarly, the term “interviewing” could invoke a formal process of eliciting a response from patients	*Ichhya badhaune paramarsha,* which conveys that healthcare providers' role is to enhance patients' motivation
Partnership	Participants believed that “patients do not have expertise” as expertise lies with the provider	*Birami sanga pani bisesh gyan huncha, which* back translates to *"patients also have specific knowledge that providers lack"*
Autonomy	Commonly believed that “sometimes family members may have to take coercive measures”	*Swatantrata* back-translates to "freedom." It implies that patients are free to make their health decisions
Compassion	No concern	*Karuna back translates to* “compassion”
Evocation	No concern	*Birami bata sikne,* which back translates to "learn from the patients." It conveys that our goal as healthcare providers is to have patients share solutions about their health
Open-ended questions	No concern	*Khula prashna sodhne,* which translates to "ask open questions"
Affirmation	Thought to be effective with patients who are most resistant to change	*Samarthan garne* back-translates to "being supportive"
Reflective listening	It was considered a useful strategy to engage with patients Often, reflections were turned into questions by using tag questions such as "right?"	*Shabdantaran garne wa doharaune* back-translates to "modifying some of the words or repeating them"
Summarize	Participants were familiar with the strategy and agreed it was helpful to keep patients engaged	*Saransha sunaune* back-translates to “convey the summary”
Change talk	No concern	*Pariwartan ka shabdaharu* back translates to “words relating to change”

## Discussion

MI carries several cultural assumptions, particularly around individual freedom and autonomy, which may not resonate in cultural settings where the family or the community’s role in health decision-making is highly valued. Our experience and findings demonstrate that although MI adaptation faces challenges in cultures where such assumptions may not be as prevalent, cross-cultural adaptation with key informant feedback can lead to creative strategies that recognize both the patient’s freedom and their role as members of a complex social fabric. Our adaptation was supported by using bilingual, bicultural clinicians and field-testing with health workers who are likely to bridge the adaptation process between researchers and front-line providers [16].

The participants’ recognition that patients' behaviors cannot be controlled and their suggestion to employ MI with various family members are examples of strategies that can help achieve this goal in non-western settings. A firm knowledge of local culture is necessary to understand how cultural components can impact a persons’ behavior [9, 26].

Although the literature on cross-cultural MI adaptation outside the US is very limited, some of our findings overlap with themes from other countries. Numerous studies mention the use of MI in their intervention, but they do not report experiences from cross-cultural adaption and have often bundled MI into other interventions [27–29]. While discussing reflective listening as core competencies of MI, we found out that the participants ended reflections as questions rather than statements. This finding is similar to the challenge faced in Thailand, where Thai counselors also tended to end reflections with upward inflections to soften the message, turning statements into questions [30]. That research group reported facing challenges while attempting to assess MI fidelity and developed a process to sort out sentences from questions that required deep and intensive cross-cultural understanding. Addressing this potential challenge early on, preferably during the training stage, may help maintain treatment fidelity.

Despite the initial hesitation, we found that the participants emphasized the need to view patients as equal partners. A study conducted among South Asians living in Canada recommends using open-ended questions to explore patients’ perspectives and understanding about medications [9]. Another study among Hispanics living in the US found that participants responded favorably to counselors who were collaborative and emphasized the social and cultural aspect of drinking [12, 31]. These findings highlight the need to work in partnership with patients across various settings, cultures, and conditions.

An important limitation is that our study site’s CHW program has been providing home-based care to patients in communities with chronic illnesses for several years prior to our study. The program has experienced challenges in employing traditional methods such as patient education and advice giving for behavior change. This may have made the participants more interested in learning MI. It is unclear if the MI concepts would be as readily acceptable to a new team that may not have experienced the limitations of MI-inconsistent approaches to behavior change.

The small sample size in the field test and not having discussions with the CHWs are important limitations of the study. However, all the five nurses at the clinic were invited and accepted to become involved in the field-test. CHWs perspectives are being captured in an ongoing study and will be disseminated in a future publication.

Adapting MI to the local context in different global settings is likely to continue to become an important research topic. As the global burden of non-communicable diseases (NCDs) has increased, the World Health Organization has released the Package of Essential Noncommunicable (PEN) guideline, which includes validated and evidence-based clinical protocols for diagnosis and treatment of NCDs in primary health care in low-resource settings. Given the prominent role of health behaviors in addressing NCDs, PEN includes MI as a core module for behavioral interventions to address key modifiable risk factors such as tobacco and alcohol use [19].

## Conclusions

Our findings from rural Nepal can help inform cross-cultural adaptations and training of MI to help providers improve care engagement among patients with chronic illness in global settings.

## Data Availability

Available from the corresponding author on request.

## Abbreviations

MI: Motivational Interviewing
CHW: Community Health Worker
OARS: Open-ended questions, affirmations, reflections, and summaries
